# Closed‐incision negative‐pressure wound therapy after Bascom's cleft lift surgery for pilonidal sinus disease: A randomized study comparing healing

**DOI:** 10.1111/codi.17198

**Published:** 2024-10-06

**Authors:** Ida Kaad Faurschou, Marlene Julia Sørensen, Allan Gorm Pedersen, Simon Ladefoged Rasmussen, Rune Erichsen, Susanne Haas

**Affiliations:** ^1^ Department of Clinical Medicine Aarhus University Aarhus Denmark; ^2^ Pilonidal Disease Center, Department of Surgery Randers Regional Hospital Randers Denmark; ^3^ Department of Surgery Horsens Regional Hospital Horsens Denmark; ^4^ Department of Clinical Epidemiology Aarhus University and Aarhus University Hospital Aarhus Denmark

**Keywords:** Bascom cleft lift, ciNPT, cleft lift surgery, negative‐pressure wound therapy, non‐healing wound, pilonidal disease, pilonidal sinus, pilonidal sinus disease, pilonidal surgery, recurrent pilonidal disease, surgical flaps, vacuum‐assisted closure

## Abstract

**Aim:**

Despite favourable outcomes in recurrence after off‐midline closure techniques in pilonidal surgery, between 18% and 40% of patients suffer from prolonged postoperative wound healing. The aim of this work was to investigate if closed‐incision negative‐pressure wound therapy (NPWT) promotes wound healing after Bascom's cleft lift (BCL) surgery for complicated pilonidal sinus disease compared with conventional drainage and dressing.

**Method:**

Patients were randomized to either NPWT for 4–7 days or loop‐vessel drain for 24 h and a dry dressing postoperatively. Healing was evaluated by a wound care nurse blinded for randomization at 2 and 12 weeks postoperatively (primary endpoint). Healing was defined as one or no closing defects of ≤5 mm and with no undermining.

**Results:**

Although we had wanted to recruit 200 patients, the study was terminated at 118 patients (NPWT group, *n* = 60; control group, *n* = 58) after interim analysis. Patients were comparable by age, sex, body mass index, previous smoking status and indication for BCL surgery. At 2 weeks 12% of patients were healed in both the NPWT and control groups [risk difference = 0.00(95% CI −0.12 to 0.11), *p* = 1.00]. After 12 weeks, 68% of patients were healed in the NPWT group and 72% in the control group [risk difference = −0.03 (95% CI 0.19 to 0.13), *p* = 0.82]. There was no significant difference in pain experienced postsurgery. In a symptom‐based questionnaire, the control group reported self‐esteem to be less affected (*p* = 0.015).

**Conclusion:**

Closed‐incision negative‐pressure wound therapy did not significantly improve healing after BCL surgery for complicated pilonidal sinus disease.

**What does this paper add to the literature?**

The treatment of complicated pilonidal sinus disease is characterized by poor healing. Negative‐pressure wound therapy (NPWT) has been successful in the management of open wounds. This study is the first randomized controlled trial to investigate the effect of closed‐incision NPWT in treatment of pilonidal sinus disease and shows that NPWT has no added benefit for wound healing after such surgery.

## INTRODUCTION

Pilonidal sinus disease (PSD) is a common disorder involving the crena ani. The estimated incidence is 48 per 100 000, although geographical variation is considerable [1, 2]. The disorder mostly affects the young, causing either acute infections or chronic manifestations with sinus or wound formation with considerable negative impact on quality of life [3].

The treatment of recurrent and complicated PSD remains controversial. The traditional surgical approach is excision of the affected tissue with primary closure or open secondary healing. Regardless of disease manifestation, this treatment is characterized by poor healing, complicated postoperative ulceration and relapse.

Bascom's cleft lift (BCL) operation is one of several lateralization techniques that have shown promising results in recent decades [4, 5, 6]. Compared with the other generally accepted surgical lateralization techniques, it has the advantage that only the affected skin is excised; subcutaneous fat is left in situ independently of its involvement. However, minor or major wound dehiscence or later arising defects with prolonged healing is a common problem and has been reported in up to 40% of BCL cases [5, 7, 8].

Negative‐pressure wound therapy (NPWT) consists of an open cell foam dressing covered in adhesive dressing attached to a vacuum pump that creates a subatmospheric pressure and thus drains away wound exudate and promotes wound healing. The system also works by altering the profile of cytokines locally acting indirectly as anti‐inflammatory agents, by increasing local growth factors and by promoting angiogenesis [9]. In the management of PSD, NPWT has been used to improve healing after excision with healing by secondary intent in case series [10, 11, 12] and a cohort study of 61 patients [13] which all showed promising results in terms of prompt wound healing and minimal complication. Two randomized controlled trials (RCTs) (19 and 49 patients) have likewise shown favourable wound healing after excision with healing by secondary intent [14, 15]. A retrospective study of 85 children compared the use of NPWT in combination with side‐swing flap with both side‐swing flap without NPWT and excision with healing by secondary intent. NWPT was an effective treatment associated with a low recurrence rate and minimal morbidity, although hospitalization was prolonged [16]. To our knowledge, however, no RCT has been performed to investigate the effect of closed‐incision NPWT in combination with off‐midline procedures for complicated PSD.

The aim of this study was to compare surgical outcomes in patients with complicated PSD undergoing BCL surgery with or without closed‐incision NPWT by assessing healing 2 and 12 weeks after surgery (primary endpoints), postoperative pain during the first 2 weeks after surgery, symptom relief after 2 and 12 weeks after surgery and recurrence at any point during the study period.

## METHOD

### Ethics and consent

The study was performed at the Pilonidal Disease Center, Department of Surgery, Randers Regional Hospital, Denmark and was conducted in conformity with the Helsinki Declaration after written informed consent. The protocol was approved by the Ethics Committee of the Central Denmark Region (1–10–72‐39‐18) and registered with clinicaltrial.gov (protocol ID RCTBCL2018). The applied NPWT system (Prevena™ incision management system) is CE marked.

### Design

The study was designed as a single‐blinded, randomized controlled superiority trial comparing healing 2 weeks postoperatively after BCL surgery with or without closed‐incision NPWT as the primary endpoint.

### Subjects

Patients were included consecutively at the Pilonidal Disease Center from January 2019 until April 2022. The Department is the regional centre for the treatment of complicated PSD. Patients were given information about the study at the initial clinic by a BCL surgeon and details included prior to surgery. Randomization was carried out by the operating surgeon preoperatively just before the patient entered the theatre according to the SNOSE principle (sequentially numbered, opaque sealed envelopes) [17].

All patients met our three standard criteria for BCL surgery: complicated disease with primary manifestations too extensive for minor surgery (Bascom's pit pick), poor postoperative healing after elective pilonidal surgery (>2 months) or recurrence after previous elective surgery for PSD.

Exclusion criteria included age <18 years, active smoker (complete cessation a minimum of 6 weeks preoperatively was accepted), patients with PSD lesions ≤3 cm from the anus (to ensure proper adherence of the NPWT dressing), patients with allergies to silver or acrylic and patients considered unable to adhere to study protocol (e.g. language difficulties, severe mental illness).

### Surgery

BCL surgery was conducted according to standard principles by one of four consultant surgeons dedicated to pilonidal surgery. Surgery was carried out under general anaesthesia with the patient in the prone position. Intravenous ciprofloxacin and metronidazole were administered at induction.

The cleft was clipped of hair and marked with the proposed area of excision including the most affected side while respecting the safety margins where the buttocks touch upon slight pressure bilaterally. Only the skin was removed, with care was taken to remove as little subcutaneous tissue as possible. Careful attention was made in cleaning any sinus tracts, hair and debris and in mobilizing fibrotic tissue. From the medial portion of the excised area a skin flap was raised on the opposite side with a thickness of 7–8 mm. and brought across the midline.

A loop‐vessel drain (control group only) was placed and the midline subcutaneous fat was sutured with PDS™ 3.0. The dermis was sutured with interrupted inverted PDS™ 3.0 sutures and the skin closed using noninterrupted Monocryl™ 3.0. sutures intradermally.

### The intervention

SNOSE randomization determined whether patients were allocated to standard postoperative wound care or NPWT. Standard treatment consisted of a loop‐vessel drain (passive) and a dry draping for 24 h. If patients were randomized to NPWT, the Prevena™ Customizable™ dressing (KCI Europe Holding BV, Utrecht, The Netherlands) was applied by the consulting surgeon directly after surgery with the patient in prone jack‐knife position and connected to a V.A.C.® Therapy Unit (V.A.C. SIMPLICITY™) at 125 mmHg. The NWPT system was removed by a local nurse or doctor at 7 days. Patients were instructed to contact the surgical clinic if there was any leakage. If the system leaked and had to be removed after <4 days, the treatment was considered a failure. After removal, patients used sanitary pads in their undergarments to absorb any secretions.

All patients received 3 days of oral antibiotics postoperatively (ciprofloxacin 250 mg × 2 per day and metronidazole 500 mg × 3 per day). All patients were instructed to refrain from strenuous activities for 6 weeks. Hair removal in the natal cleft was carried out twice a week by a nurse or by a designated family member following instructional guidance provided through a departmental instructional video.

### Outcome

At 2 weeks and 12 weeks after surgery, patients were seen by a specialized wound care nurse who was blinded to the randomization allocation. Healing was assessed according to standardized definitions: healing was considered successful if no undermining of the cicatrices was present and there was no more than one defect which was ≤5 mm. If healing was not achieved, the depth of the defect was measured and classified as superficial (maximum 5 mm) or deep (≥5 mm). If healing had not occurred at 2 weeks the wounds were treated according to department standards with increased wound care by the nurse supervised by the operating surgeon. Patients healed at 2 weeks but subsequently lost to follow‐up were defined as healed at 12 weeks.

### Prolonged healing and recurrence

Prolonged healing was defined as lack of healing ≥12 weeks after BCL surgery. At study end, the database was examined to uncover if the patients had received further surgical treatment. If a patient received one or more procedures after the initial operation, their medical record was examined to distinguish between prolonged healing or recurrence. Recurrence was defined as recurrent disease, when diagnostic criteria were met after previous complete wound healing objectified by physician or wound care nurse.

### Pain diary

During the first 2 weeks after surgery, patients evaluated pain daily using a visual analogue scale score.

### PSD symptom‐based questionnaire

All patients answered a disease‐specific symptom‐based questionnaire at baseline and 2 and 12 weeks postoperatively. This questionnaire had previously been used in our group to uncover the impact of PSD and examine symptoms (odour, secretion and pain) and everyday influences (uncertainty regarding disease behaviour, self‐esteem, the ability to be intimate with others, ability to participate in leisure activities and the overall affect on quality of life) [3]. Each question has a symptoms score ranging from 0 to 5 and a possibility of answering ’I don't know’ (Table S3).

### Power and sample size

Based on clinical experience, it was estimated that 75% of patients would heal primarily. The least clinically relevant difference between standard treatment and NPWT was set to 15%. With a significance (*α*) level of 5% and power (*β*) of 80%, sample size turned out to be 100 patients in each treatment arm, i.e. a total of 200 patients. Because the assumptions behind this power calculation were uncertain, an interim analysis was planned by an independent body. The interim analysis was scheduled to be performed after the first 100 patients had completed the 2‐week assessment. This analysis would enable any decision to terminate the trial early due to futility or superiority and to re‐estimate the sample size. As the area treated with NPWT in this study is curved and close to the anus with innate challenges in terms of adherence of the dressing it was decided that a treatment failure rate greater than 15% would result in the study being discontinued as we would not be able to assess for inferior or superiority. The study was conducted using an intention‐to‐treat approach, and thus included those patients with NPWT treatment failure.

### Statistics

Continuous data are presented as means with standard deviations (SD) or as median with interquartile range (IQR) and compared with either the two‐sample *t*‐test or Mann–Whitney test according to distribution. Categorical data are presented as frequencies with percentages and analysed using Fisher's exact test. Risk difference is shown with a 95% confidence interval (95% CI). A *p*‐value <0.05 was considered statistically significant. Statistical analyses were performed with STATA software, version 17.0 (STATA, StataCorp, College Station, Texas, USA). Graphs were designed in Microsoft Excel (Microsoft 365).

## RESULTS

**FIGURE 1:**
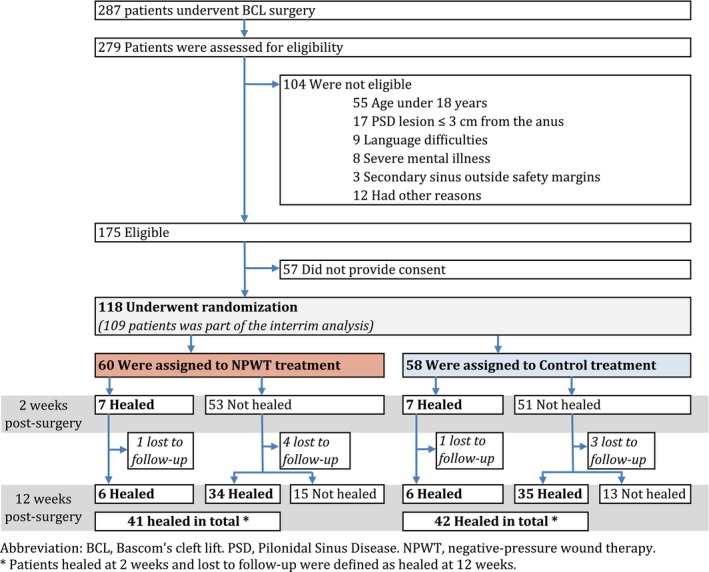
Flowchart of study enrolment, randomization and healing 2 and 12 weeks postsurgery in the negative‐pressure wound therapy (NPWT) group and the control group. BCL, Bascom's cleft lift surgery; PSD, pilonidal sinus disease. *Patients healed at 2 weeks and lost to follow‐up were defined as healed at 12 weeks.

In the study period, 287 patients underwent BCL surgery. Of these, 175 were eligible candidates and 118 consented to participate in the study (Figure 1).

The interim analysis was performed using data from the initial 109 patients, leading to premature termination of the study as it was concluded it was highly unlikely that NPWT would be superior to standard treatment. Study inclusion continued during the interim analysis, including an additional nine patients before the study was terminated. The results presented are based on these 118 patients, where 60 patients had been randomized to NPWT and 58 patients to standard postoperative wound care (Figure 1).

Patients were comparable in baseline characteristics (Table 1). The median operation time was 55 min (IQR 50–67 min) in the NPWT group compared with 50 min (IQR 45–55 min) in the control group.

**TABLE 1:** Baseline characteristics of patients in the negative‐pressure wound therapy (NPWT) group and the control group.

	NPWT (*n* = 60)	Control (*n* = 58)	*p*‐value
Age (years), median (IQR)	24 (21–29)	25 (21–29)	0.651^a^
BMI (kg/m^2^), mean (SD)	27.4 (4.7)	27.4 (4.0)	0.924^b^
Sex			
Female, *n* (%)	12 (20%)	6 (10%)	0.201^c^
Male, *n* (%)	48 (80%)	52 (90%)	
ASA grade			
1, *n* (%)	47 (81%)	43 (77%)	0.567^c^
2, *n* (%)	11 (19%)	11 (20%)	
3, *n* (%)	0 (0%)	2 (4%)	
Diabetes mellitus, *n*	1	1	
Immunocompromised, *n*	0	1	
Hidradenitis suppurativa, *n*	2	0	
Smoking status			
Never smoker, *n* (%)	40 (67%)	34 (59%)	0.45^c^
Previous smoker, *n* (%)^d^	20 (33%)	24 (41%)	
Months since symptom onset, median (IQR)	24 (9–48)	25 (11–60)	0.686^a^
Indication for BCL surgery			
Primary extensive manifestation, *n* (%)	18 (30%)	20 (34%)	0.80^c^
Nonhealing wounds after surgery, *n* (%)	22 (37%)	18 (31%)	
Recurrence after elective surgery, *n* (%)	20 (33%)	20 (34%)	

Abbreviations: ASA, American Society of Anesthesiologists; BCL, Bascom's cleft lift; BMI, body mass index; IQR, interquartile range.

^a^ Mann–Whitney test.

^b^ Student's *t*‐test.

^c^ Fisher's exact test.

^d^ All current smokers quit smoking min 6 weeks prior‐ and post‐surgery.

### Healing

Seven (12%) patients achieved complete healing at 2 weeks postoperatively in the NPWT group compared with seven (12%) in the control group, with a risk difference of 0.00 (95% CI −0.12 to 0.11) (*p* = 1.00) (Table 2). At 12 weeks postoperatively, 41 (68%) of patients were completely healed in the NPWT group versus 42 (72%) in the control group with a risk difference of −0.03 (95% CI −0.19 to 0.13) (*p* = 0.82). Overall, a total of 28 (25%) patients did not achieve complete healing within 12 weeks (Figure 1).

**TABLE 2:** Number of patients healed at 2 and 12 weeks, suffering from prolonged healing or recurrent disease in the negative‐pressure wound therapy (NPWT) group and the control group.

	NPWT group (*n* = 60), *n* (%)	Control group (*n* = 58), *n* (%)	Risk difference (95% CI)	Test for significance *p*
Healed^a^ at *t* = 2 weeks	7 (12%)	7 (12%)	0.00 (−0.12 to 0.11)	1.000^b^
Healed^a^ at *t* = 12 weeks	41 (73%)	42 (76%)	−0.03 (−0.19 to 0.13)	0.828^b^
Prolonged healing^c^	15 (26%)	13 (24%)	0.03 (−0.13 to 0.18)	0.089^b^
Further surgical treatment needed due to prolonged healing	6 (10%)	5 (9%)		
Recurrence (varying follow‐up time)^d^	4 (7%)	1 (2%)		
Additional use of antibiotics	1 (2%)	1 (2%)		

^a^ Healing was defined as (1) no undermining of the cicatrices was present and (2) one or no closing defects of ≤5 mm.

^b^ Fisher's exact test.

^c^ Prolonged healing was defined as lack of healing ≥12 weeks after Bascom's cleft lift surgery.

^d^ Recurrence was defined as recurrent disease when diagnostic criteria were met after previous complete wound healing objectified by a physician or wound care nurse.

Subgroup analysis according to indication for BCL surgery did not show any statistically significant difference in healing between treatment groups (Table S1).

At 2 weeks, the NPWT group had 29 patients with superficial defects (<5 mm depth) and 23 with deep defects (>5 mm depth). The control group had 31 patients with superficial defects and 19 patients with deep defects (*p* = 0.552). At 12 weeks, we observed eight patients with superficial defects and seven patients with deep defects in the NPWT group compared with six with superficial defects and seven with deep defects in the control group (*p* = 1.000) (Table S2).

### Treatment failure and loss to follow‐up

Five patients (8%) were lost to follow‐up between 2 and 12 weeks in the NPWT group and four patients (7%) in the control group. NPWT failed in two patients, where one of them was healed at 12 weeks postsurgery and one was lost to follow‐up. No other adverse events were observed.

### Prolonged healing and recurrence

Eleven patients (six in the NPWT group and five controls) with prolonged healing needed further surgical treatment. During the study period, four patients in the NPWT group presented with recurrent PSD and only one patient in the control group, giving a total early recurrence rate—regardless of group—of 4.6% with a median time to recurrence of 222 days (IQR 210–494 days) (Table 2).

### Questionnaires

#### Pain dairy

**FIGURE 2:**
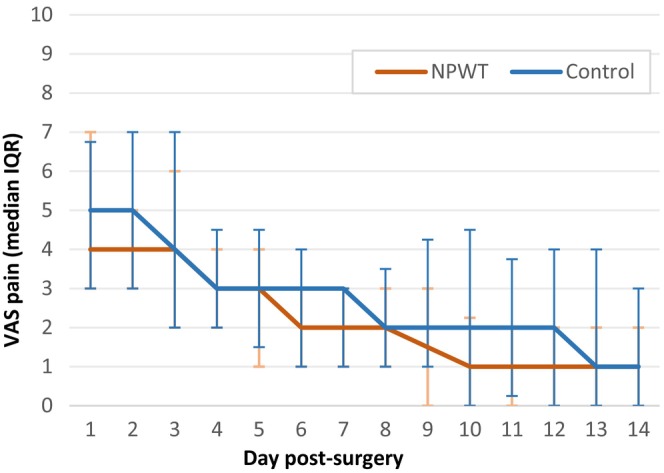
Median pain reported on a Visual Analogue Scale (VAS) the first 14 days after surgery in the negative‐pressure wound therapy (NPWT) group and the control group. In both groups the pain reported decreased steadily with no significant difference.

The pain diary had a response rate of 64%. On day 1, the median pain level was 4 (1–9) in the NPWT and 5 (1–9) in the control group (*p* = 0.913). The median pain levels gradually decreased to 1 (1–7) in the NPWT group and 1 (1–10) in the control group by day 14, with no significant difference observed between the groups (*p* = 0.488) (Figure 2).

#### PSD symptom‐based questionnaire

The symptom‐based questionnaire was answered before surgery (response rate 98%) and at 2 weeks (response rate 90%) and 12 weeks (response rate 71%) after surgery. The groups were comparable at baseline. At 12 weeks, the control group reported to be less affected in self‐esteem [median 0 (IQR 0–1)] compared with the NPWT group [median 0 (IQR 0–0.25)] (*p* = 0.015), where nine patients had a value of 2 (‘Yes, and it's annoying but not daily’) or above compared with two patients in the control group. Apart from that, there was no significant difference between the groups and the symptoms were decreasing significantly over time as healing occurred (Table S3).

## DISCUSSION

We present results from a RCT comparing healing in patients with PSD undergoing BCL surgery with or without closed‐incision NPWT. Healing at 2 and 12 weeks showed no difference between the groups. The study was prematurely terminated based upon interim analysis that found it very unlikely that the inclusion of an additional 100 patients could result in NPWT treatment appearing better than the standard treatment at a level listed as the smallest clinically significant difference. Additionally, our power calculation was based on an expectation of a spontaneous healing rate of approximately 75% and that the minimum clinically significant difference (‘difference’) in the treatments was set to 15%. The interim analysis revealed that the healing after 2 weeks and 12 weeks was 12.5%–14.6% and 58.2%–66.1%, respectively, far from matching the expectations set when planning the study.

To our knowledge this is the first RCT to evaluate the effect of closed‐incision wound management with NWPT on healing in complicated PSD. Previous studies on NPWT in the treatment of PSD have primarily included patients after excision of PSD leaving open wounds to heal by secondary intent, and so cannot be compared with our study [10, 11, 12, 13, 14, 15]. Dorth et al. compared three groups of children undergoing PSD surgery: open excision (*n* = 48), flap (*n* = 15) and hybrid (flap and NPWT, *n* = 22). Even though this study reported that flap in combination with NPWT was superior to the two other groups combined, a comparison of only flap and flap with NPWT revealed no significant difference and supports our findings [16].

In our study, we gave special attention to precisely defining endpoints: healing after surgery with primary closure, prolonged healing and recurrence. Previous surgical techniques for PSD have been evaluated using recurrence as the sole endpoint with recurrence often confused with prolonged healing [18, 19]. We recognize that in our study prolonged healing and recurrence are high. This may be because only patients with complicated PSD were recruited and because we adhered to a conservative definition of healing. We therefore believe that our results are in line with other studies where the definition of healing is obscure or absent [5, 8, 20].

We had some concerns about the feasibility of the study due to the curved wound and near anal location increasing the risk of NWPT leakage. The application of this appliance was meticulously performed under optimal conditions by the operating surgeon with a treatment failure rate of two out of 60 patients. Our study also refutes concerns raised that NPWT would increase postoperative pain compared with an ordinary dressing regime [21]. Our study is not without limitations. Firstly, we incorporated interim analysis to terminate the study if necessary, as previously discussed. Our concern about NPWT revolved around two factors: the rate of treatment failure (especially given the unconventional application of NPWT to curved or irregular wound areas) and our assumptions regarding healing dynamics. Additionally, we were concerned about the added cost of the equipment, and from the patient's point of view the inconvenience of carrying the V.A.C. pump and always maintaining the negative pressure.

Despite the cessation of the study before full recruitment, this is still a reasonably sized study with 118 patients recruited and a convincing negative result.

This study while blinded for the clinical assessment could not be blinded to the patients. This inability to conduct a double‐blind study in which patients are unaware of the intervention being administered may lead to bias in patients' perception of symptoms. This did not affect the evaluation of the primary endpoint as it was performed by a specialized wound care nurse who was blinded to the randomization process. We do acknowledge the risk of treatment disclosure during follow‐up. However, as results are not specifically favouring any treatment, this bias is likely to be minor. The symptom‐based questionnaire could also be influenced by bias in perception. Patients may exhibit a preference for modern treatment options and can be influenced by the perspectives of healthcare personnel on different wound dressing options.

Other factors to consider include smoking status where we relied on patient disclosure and an agreement to refrain from or stop smoking. Undisclosed smoking, which may have impacted healing, cannot be ruled out [22, 23].

The use of a wound drain to prevent the development of seroma in the control group is an inherent component of the BCL procedure. It was unfeasible to incorporate a drain beneath the NPWT device as it significantly increased the risk of leakage. Since NPWT also drains away wound exudate we did not think this would significantly disadvantage the NPWT cohort.

Lastly, we examined recurrence as a secondary not primary outcome, as we did not expect NWPT to influence this outcome. As such, a planned follow‐up was not prioritized during the study design phase and so recurrence was assessed at various and not specific points in time.

## CONCLUSION

This RCT showed no significant difference in healing when closed‐incision NPWT was applied after BCL surgery for complicated PSD. The persistent issue of poor wound healing is a common problem among individuals with PSD that necessitates ongoing clinical and developmental attention. On the basis of the interim analysis the study was discontinued as a clinically relevant improvement in treatment seemed unlikely.

## AUTHOR CONTRIBUTIONS

**Ida Kaad Faurschou:** Writing – original draft; writing – review and editing; visualization; formal analysis; data curation. **Marlene Julia Sørensen:** Conceptualization; project administration; methodology; funding acquisition; writing – review and editing; investigation. **Allan Gorm Pedersen:** Conceptualization; methodology; project administration; supervision; writing – review and editing. **Simon Ladefoged Rasmussen:** Data curation; formal analysis; writing – review and editing. **Rune Erichsen:** Supervision; writing – review and editing; methodology. **Susanne Haas:** Conceptualization; investigation; funding acquisition; writing – review and editing; supervision; resources; project administration; methodology.

## FUNDING INFORMATION

This study was initiated by the Pilonidal Disease Center at Randers Regional Hospital, Denmark. The authors of the study did not receive payment; however, the Prevena™ NPWT systems were sponsored by the producer of the products, Acility.

## CONFLICT OF INTEREST STATEMENT

The authors declare no conflict of interests.

## ETHICS APPROVAL STATEMENT

The study was conducted in conformity with the Helsinki Declaration after written informed consent. The protocol was approved by the Ethics Committee of the Central Denmark Region (1‐10‐72‐39‐18).

## CLINICAL TRIAL REGISTRATION

Registered with clinicaltrial.gov (protocol ID RCTBCL2018).

## Supporting information


Table S1.

Table S2.

Table S3.

## Data Availability

The data that support the findings of this study are available from the corresponding author upon reasonable request.
