# Supplementary material for: Closed‐incision negative‐pressure wound therapy after Bascom's cleft lift surgery for pilonidal sinus disease: A randomized study comparing healing
Source: Colorectal Dis. 2024 Oct 6;27(1):e17198. doi: 10.1111/codi.17198 (PMC11683170; doi:10.1111/codi.17198)
Supplement: Supplementary file 1 — Table S1. [file CODI-27-0-s001.docx]

**Supplementary table 1:** Heling based on indication for BCL surgery. Number of patients healed at 2 and 12 weeks in the NPWT (negative-pressure wound therapy) group and the control group when subdivided into groups based on indication for BCL surgery.

|  | **NPWT group**  (n= 60) | | **Control group**  (n=58) | | Test for significance |
| --- | --- | --- | --- | --- | --- |
| ***2 weeks*** | | | | | |
|  | *n(total)* | *Healed, n(%)* | *n(total)* | *Healed, n(%)* |  |
| Primary extensive | 18 | 2 (11%) | 20 | 3 (15 %) | *p = 1.00^3^* |
| Non-healing wounds after surgery | 22 | 2 (10%) | 18 | 0 (0%) | *p = 1.00^3^* |
| Recurrence after elective surgery | 20 | 3 (15%) | 20 | 4 (20%) | *p = 0.50^3^* |
| Test for significance based on indication group (NPWT and Control group pooled) | | | | | *p = 0.22^3^* |
| ***12 weeks*** | | | | | |
| Primary extensive | 18 | 11 (73%) | 20 | 14 (74%) | *p = 1.00^3^* |
| Non-healing wounds after surgery | 22 | 16 (73%) | 18 | 11 (65%) | *p = 0.73^3^* |
| Recurrence after elective surgery | 20 | 14 (74%) | 20 | 17 (89%) | *p = 0.41^3^* |
| Test for significance based on indication (NPWT and Control group pooled) | | | | | *p = 0.46^3^* |

^3^Fisher’s exact test.
