# Supplementary material for: Closed‐incision negative‐pressure wound therapy after Bascom's cleft lift surgery for pilonidal sinus disease: A randomized study comparing healing
Source: Colorectal Dis. 2024 Oct 6;27(1):e17198. doi: 10.1111/codi.17198 (PMC11683170; doi:10.1111/codi.17198)
Supplement: Supplementary file 2 — Table S2. [file CODI-27-0-s002.docx]

**Supplementary table 2:** Distribution between deep and superficial defects measured at 2 and 12 weeks in not healed patients in the NPWT (negative-pressure wound therapy) group and the control group.

|  | **NPWT group**  (n= 60) | **Control group** (n=58) | Test for significance |
| --- | --- | --- | --- |
|  | *2 weeks* | | |
| Superficial defect (max 5 mm), n(%) | 29 (56%) | 31 (62%) | *p = 0.552^3^* |
| Deep defect (>5 mm), n(%) | 23 (44%) | 19 (38%) |  |
|  |  |  |  |
|  | *12 weeks* | | |
| Superficial defect (max 5 mm) | 8 (53%) | 6 (46%) | *p = 1.000^3^* |
| Deep defect (>5 mm) | 7 (47%) | 7 (54%) |  |
|  |  |  |  |

^3^Fisher’s exact test.
