# Supplementary material for: Closed‐incision negative‐pressure wound therapy after Bascom's cleft lift surgery for pilonidal sinus disease: A randomized study comparing healing
Source: Colorectal Dis. 2024 Oct 6;27(1):e17198. doi: 10.1111/codi.17198 (PMC11683170; doi:10.1111/codi.17198)
Supplement: Supplementary file 3 — Table S3. [file CODI-27-0-s003.docx]

**Supplementary table 3:** Pilonidal sinus disease specific symptoms pre-surgery, 2 weeks and 12 weeks post-surgery based on PSD Symptom-based questionnaire with symptoms score ranging from 0-5 and a count of patients answering “I don’t know”. Median score (IQR) for each symptom is shown at each timepoint in both the NPWT (negative-pressure wound therapy) group and the control group.

|  | **NPWT** | | | **Control** | | |  |
| --- | --- | --- | --- | --- | --- | --- | --- |
| Symptoms | Symptom score*, median (IQR) | |  | Symptom score*, median (IQR) | |  |  |
|  |  |  | *"I don't know",*  *(n)* |  |  | *"I don't know",*  *(n)* | *Test for significance^3^* |
|  | *Pre-surgery* | | | | | |  |
| Smell | 2 | (1-3) | *5* | 1 | (0-3) | *5* | *0.560* |
| Secretion | 3 | (1.5-3) | *0* | 3 | (2-3) | *1* | *0.471* |
| Pain | 2 | (1-3) | *0* | 2 | (2-3) | *0* | *0.840* |
| Uncertainty | 2 | (1-3) | *9* | 2 | (1-3) | *4* | *0.270* |
| Self-worth | 2 | (0-3) | *6* | 2 | (0-3) | *3* | *0.548* |
| Intimacy | 1 | (0-3) | *5* | 2 | (0-2) | *3* | *0.395* |
| Leisure | 1 | (0-2) | *2* | 2 | (0-4) | *2* | *0.469* |
| Life quality | 2 | (0-3) | *5* | 2 | (1-3) | *2* | *0.715* |
|  | *2 weeks* | | | | | |  |
| Smell | 0.5 | (0-1) | *4* | 1 | (0-2) | *0* | *0.711* |
| Secretion | 1 | (0.75-3) | *0* | 2 | (1-3) | *0* | *0.090* |
| Pain | 1 | (1-2) | *8* | 1 | (1-3) | *0* | *0.469* |
| Uncertainty | 0 | (0-2) | *11* | 1 | (0-2) | *4* | *0.719* |
| Self-worth | 0 | (0-2) | *5* | 1 | (0-2) | *2* | *0.232* |
| Intimacy | 0 | (0-2) | *8* | 0.5 | (0-2) | *4* | *0.586* |
| Leisure | 2 | (0-4) | *5* | 2 | (1-4) | *1* | *0.130* |
| Life quality | 1 | (0-2) | *3* | 1 | (0-3) | *1* | *0.533* |
|  | *12 weeks* | | | | | |  |
| Smell | 0 | (0-0.5) | *0* | 0 | (0-0) | *1* | *0.093* |
| Secretion | 0 | (0-1) | *1* | 0 | (0-1) | *0* | *0.944* |
| Pain | 0 | (0-1) | *7* | 0 | (0-1) | *0* | *0.971* |
| Uncertainty | 0 | (0-1) | *8* | 0 | (0-0) | *2* | *0.673* |
| Self-worth | 0 | (0-1) | *3* | 0 | (0-0.25) | *0* | ***0.015*** |
| Intimacy | 0 | (0-1.75) | *3* | 0 | (0-0) | *1* | *0.064* |
| Leisure | 0 | (0-1.25) | *3* | 0 | (0-1) | *0* | *0.902* |
| Life quality | 0 | (0-1) | *6* | 0 | (0-1) | *0* | *0.992* |

* Symptoms-score: 0 = No, never, 1 = Yes, but it’s not annoying, 2 = Yes, and it’s annoying but not daily, 3 = Yes, and it’s annoying and it’s daily, 4 = Yes, and it affects me in my daily activities, 5 = Yes, and it makes my daily activities impossible to do

^3^Fisher’s exact test.
